# Putting Temperature into the Equation: Development and Validation of Algorithms to Distinguish Non-Wearing from Inactivity and Sleep in Wearable Sensors

**DOI:** 10.3390/s22031117

**Published:** 2022-02-01

**Authors:** Sara Pagnamenta, Karoline Blix Grønvik, Kamiar Aminian, Beatrix Vereijken, Anisoara Paraschiv-Ionescu

**Affiliations:** 1 Ecole Polytechnique Federale de Lausanne (EPFL), Laboratory of Movement Analysis and Measurement (LMAM), CH-1015 Lausanne, Switzerland; sara.pagnamenta@alumni.epfl.ch (S.P.); kamiar.aminian@epfl.ch (K.A.); 2 Department of Neuromedicine and Movement Science, Norwegian University of Science and Technology, N-7491 Trondheim, Norway; karoline.b.gronvik@ntnu.no (K.B.G.); beatrix.vereijken@ntnu.no (B.V.)

**Keywords:** activity monitoring, wearable devices, non-wearing time, accelerometer, temperature sensor, event-based detection algorithms

## Abstract

Long-term monitoring of real-life physical activity (PA) using wearable devices is increasingly used in clinical and epidemiological studies. The quality of the recorded data is an important issue, as unreliable data may negatively affect the outcome measures. A potential source of bias in PA assessment is the non-wearing of a device during the expected monitoring period. Identification of non-wear time is usually performed as a pre-processing step using data recorded by the accelerometer, which is the most common sensor used for PA analysis algorithms. The main issue is the correct differentiation between non-wear time, sleep time, and sedentary wake time, especially in frail older adults or patient groups. Based on the current state of the art, the objectives of this study were to (1) develop robust non-wearing detection algorithms based on data recorded with a wearable device that integrates acceleration and temperature sensors; (2) validate the algorithms using real-world data recorded according to an appropriate measurement protocol. A comparative evaluation of the implemented algorithms indicated better performances (99%, 97%, 99%, and 98% for sensitivity, specificity, accuracy, and negative predictive value, respectively) for an event-based detection algorithm, where the temperature sensor signal was appropriately processed to identify the timing of device removal/non-wear.

## 1. Introduction

Wearable devices, including accelerometers, are the most commonly used tools to objectively evaluate physical activity and mobility in large-scale studies [1]. They measure body acceleration in all three axes, and the gathered data can be used to classify different movement behaviors and body postures [1,2,3,4]. One of the first steps when carrying out such analyses is the detection of non-wear periods, i.e., the time during which the device is not worn for various reasons, e.g., during showering or other aquatic activities, forgetting to reattach the device afterwards, or removal of the sensor due to the fact of discomfort [2,5]. The identification of non-wear periods is crucial in clinical and epidemiological studies where accelerometer-derived movements and physical activity parameters are used as health outcomes. First, detection of wear/non-wear allows for measurement of the patients’ compliance along with validation of monitoring days for further analysis [2,4]. Secondly, it reduces the risk of potential bias of derived physical activity parameters and outcome evaluation, as weak acceleration signals during non-wearing are often misclassified as sedentary behaviors or sleep by physical activity analysis algorithms. This can be particularly problematic for older adults or groups of patients that spend a significant amount of time being inactive.

The accurate detection of non-wear time is still an ongoing issue, since this problem has received little attention and studies have failed to address it adequately. Current algorithms are often based exclusively on data collected by accelerometer sensors incorporated into monitoring devices [2,4,6]. Typical methods used to detect periods of zero-activity counts, thus indicating low levels of activity, with an appropriate threshold on the activity counts signal (time series) over epochs of 30 s or 1 min. Once the entire signal is scanned, the epochs are grouped into windows of 30, 60, or 90 min in order to ignore motion artifacts [4,7]. However, such a metric presents several limitations. A “count” is an arbitrary value that is specific to the device manufacturer and, thus, difficult to generalize across different accelerometers [3,5,7]. Moreover, these algorithms are typically validated in a laboratory with devices being worn during waking hours only, meaning they cannot be applied to protocols monitoring patients in a free-living setting over a period of multiple days [5]. Finally, they are problematic in two situations in particular: when motion artifacts are present during non-wearing periods and when inactivity extends for long periods. In the first case, non-wear may be misclassified as low activity, whereas in the second case, sedentary behaviors could be misclassified as non-wear [3,7].

Few studies have focused on analyzing raw accelerometer data. Raw data are more meaningful and interpretable than activity counts, as it is the wearer of the device that directly causes the signal [3]. Ahmadi et al. [5] evaluated the performances of four algorithms based on raw data, where the standard deviation of the acceleration in three axes and the standard deviation of the vector magnitude were two of the metrics used. The algorithm using the norm of the acceleration was the most promising and was shown to be the most sensitive to movement and the most robust to noise. However, the accelerometer is sensitive to movement artifacts, independent of the used metrics, and the choice of the window that regroups shorter non-wear epochs is the determining factor. Moreover, differentiating between sleep and non-wear periods remains difficult, and misclassification is therefore unavoidable [3].

To improve the performance of non-wear algorithms, more recent studies have started to use temperature sensors. When worn in contact with the body, the device is influenced by body temperature, and once removed, the temperature signal normally decreases to reach an equilibrium with the surroundings. Two previous studies [3,8] used a temperature threshold and the average value of the temperature over a certain window to estimate non-wear periods with some differences. Zhou et al. [3] used the GENEActiv watch worn at the wrist and combined acceleration and temperature signals to identify non-wear periods, and the temperature threshold was fixed at 26 °C. Duncan et al. [8] used a dual-accelerometer system with Axivity devices worn at the thigh and lower back, and used the temperature signal alone with a dynamic threshold (computed from 18 °C) to characterize non-wear periods. The common issue with both methods is that the threshold is defined in terms of *absolute* temperature, which reduces generalizability of the results, as this value depends on the outdoor temperature, which varies among regions and on the device used. It is therefore necessary to adapt it to different situations [3,8].

Despite rapid developments in sensor technology and related software, the accurate distinction between non-wearing, daily inactivity, and sleep remains unsolved, which is particularly problematic for the long-term monitoring of frail older adults or patients with severe conditions. Therefore, the objective of the current study was to (1) develop robust non-wearing detection algorithms based on acceleration and/or temperature that reduce misclassification errors; (2) validate the algorithms using real-world data recorded according to an appropriate measurement protocol.

## 2. Materials and Methods

### 2.1. Data Collection

**Figure 1:**
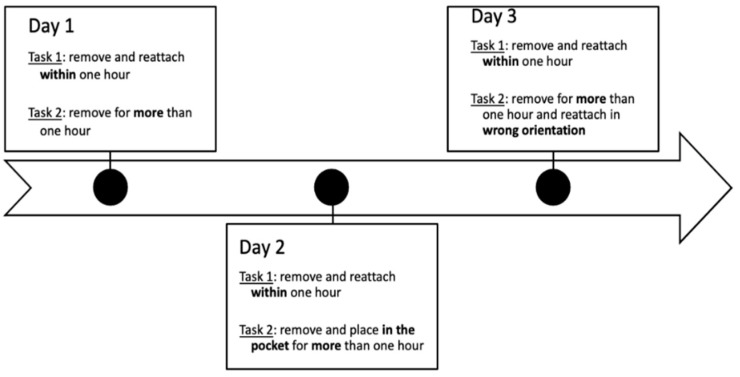
Summary of the tasks included in the data acquisition protocol.

The study included 21 healthy volunteers (13 females, 8 males, aged 24–60 years) enrolled at the Norwegian University of Science and Technology (NTNU), Trondheim, between September–November 2020. The participants were students, employees, and acquaintances, who all signed a consent form before participating. The study was assessed by the Norwegian Centre for Research Data (NSD) and conducted in accordance with the Declaration of Helsinki. Participants were monitored continuously for three consecutive days, 24 h per day, using the Axivity device AX3 (https://axivity.com/, accessed on 7 January 2021) which contains a 3-axis accelerometer and a temperature sensor. According to Axivity specifications, the temperature sensor gives a rough sense of the device’s environmental temperature. When worn, the device was fixed onto the skin at the lower back (L5) with a hypoallergenic adhesive film. Sensor data were recorded continuously during the monitoring period at a 100 Hz sampling rate. The participants were given a set of instructions for the wear and non-wear periods and were asked to complete a diary keeping detailed track of the removal periods. The protocol included two tasks per day and four different tasks in total that were intended to test the non-wear detection algorithms in different conditions as illustrated in Figure 1.

The participants were instructed to remove the device twice a day. Task 1 was performed each day and consisted of removing the device for less than an hour to test the ability of the algorithms to detect short non-wear periods, which are usually ignored by many state-of-the-art methods. The second task was different each day and consisted of removing the device for more than one hour (day 1), removing and placing the device in a pocket (day 2), and removing and reattaching the device in the wrong orientation (day 3) as indicated in Figure 1. The participants were asked to perform three claps with the sensor upon removal of the device as well as prior to reattachment to allow for comparison between diary entry times and device times.

In the diary, the participants entered the exact times of removal and reattachment for the different tasks. These times were essential to generate a reference (ground truth) for validation of the classification algorithms. Information regarding the place where the devices were stored, room and external temperatures, and bedtime (sleep) was also provided in the diary. Across participants, the diary entries showed that the inside temperature varied between 18 and 23 °C, and the outside temperature between −3 and 17 °C.

### 2.2. Developed Algorithms

Different algorithms were developed to assess the efficiency of our methodology. To test the performances of the newly developed algorithms, alternative approaches derived from the literature and based on temperature or accelerometer signals were implemented and compared.

#### 2.2.1. Accelerometer Based

The accelerometer-based algorithm, derived from Ahmadi et al., is based on the standard deviation of the vector magnitude (VM), which was preferred over the standard deviation of the acceleration signal on individual axes due to the lower sensitivity to noise and to the orientation of the device [5]. The dynamic component of VM (i.e., gravity removed) was filtered using a bandpass 4th order Butterworth filter (lower cut-off frequency fc1 = 0.5 Hz, upper cut-off frequency fc2 = 20 Hz). The standard deviation was computed at a 1 min window length and compared to an appropriate threshold, thr = 13 mg, selected according to recommendations in the literature to take into account the noise associated with the electrical components [2,7]. In accordance with the literature, non-wear periods of less than 30 min were ignored and classified as wear to avoid errors due to the motion artifacts [5]. At the end of this procedure, a binary vector was generated, labeling wear periods with 0 and non-wear periods with 1.

#### 2.2.2. Temperature Based

Depending on the methodological approach, the algorithms using the temperature sensor data were categorized as window-based or event-based. The first category includes state-of-the-art algorithms implemented in order to compare the performances of the newly developed event-based algorithms. For both approaches, the recorded temperature data are first downsampled by a factor of 6 (i.e., keeping only every 6th sample). This choice of the sampling frequency was based on the thermal response of the 20001942G temperature sensor (Microchip Technology, Linear Active Thermistor Integrated Circuit) included in the Axivity device. The steady state of the air-to-fluid bath experiment is nearly reached after ~6 s (Low-Power Linear Active Thermistor ICs MCP97009700A and MCP97019701A Datasheet. https://www.rfglobalnet.com/doc/low-power-linear-active-thermistor-ics-mcp-a-and-mcp-a-datasheet-0001, (accessed on 7 January 2021)). The signal was therefore downsampled in order to eliminate noisy fluctuations. The sampled signal was further smoothed by applying a Savitzky–Golay filter, chosen on the basis that this smoothing filter is better suited to preserve sudden changes in the signal.

##### Window-Based Approach

**Figure 2:**
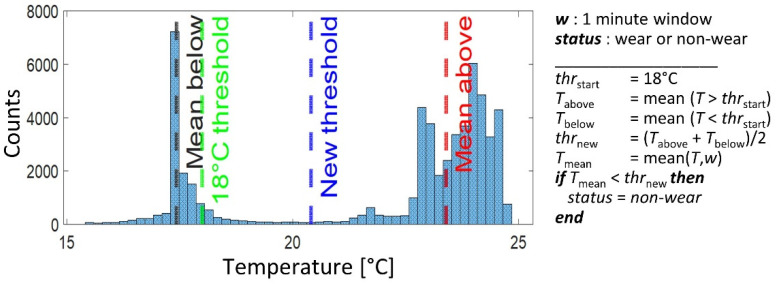
Illustrative example of the temperature (T) distribution of data recorded from one participant and pseudocode for dynamic threshold (thr) computation.

Two window-based approaches derived from Duncan et al. [8] were implemented and compared. In the first method, a dynamic threshold set around 18 °C was used to characterize wear and non-wear periods, as proposed in [8]. In the second method, we developed a dynamic/adaptive threshold approach, derived as follows. The mean of the temperature above and below 18 °C was computed and the threshold was adjusted to be in the middle of these average values as depicted in the histogram in Figure 2. The signal was then scanned with a 1 min window length, the mean of the temperature was computed, compared to the threshold and—if lower—the window was classified as non-wear (see pseudocode in Figure 2).

##### Event-Based Approach

**Figure 3:**
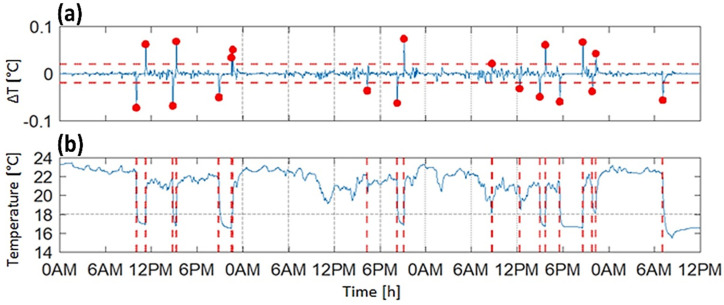
Illustrative example of the processing of the temperature signal using the event-based approach derivative (**a**) of the filtered and downsampled temperature signal (**b**) recorded during the monitoring period. The red, dashed vertical lines indicate potential non-wear events, corresponding to the timing of the red dots (detected peaks) on the signal derivative. The red, dashed horizontal lines in the upper panel (**a**) represent the ±0.02 °C thresholds used for peak detection.

Event-based algorithms look for a particular episode in the signal that is characteristic of the class one wants to identify. Currently, there is no evidence of the use of such an approach in the context of non-wear detection. In the case of the temperature signal, an appropriate event for classification would be the temperature difference between consecutive datapoints. Larger changes in the temperature occur when the sensor is removed (the temperature decreases) or reattached (the temperature increases). The derivative of the temperature signal highlights those changes by prominent peaks, compared to smaller variations due to, for example, the fact of environmental temperature fluctuations. However, due to the slower response time, the derivative signal had a relatively small amplitude, on the order of 0.01–0.02 °C (see Figure 3a). Consequently, it was difficult to apply a meaningful threshold to identify peaks directly from the derivative signal. Therefore, we adopted a detection-classification procedure [9], where, first, all candidate peaks potentially related to wear/non-wear transitions were detected using an arbitrary low threshold set at 0.02 °C. Once all the candidates were detected (Figure 3a), the classification was performed based on the changes in temperature levels around the timing of each candidate peak (Figure 3b). If the temperature difference was large enough (≥3 °C), the respective peak was retained for the classification stage.

**Figure 4:**
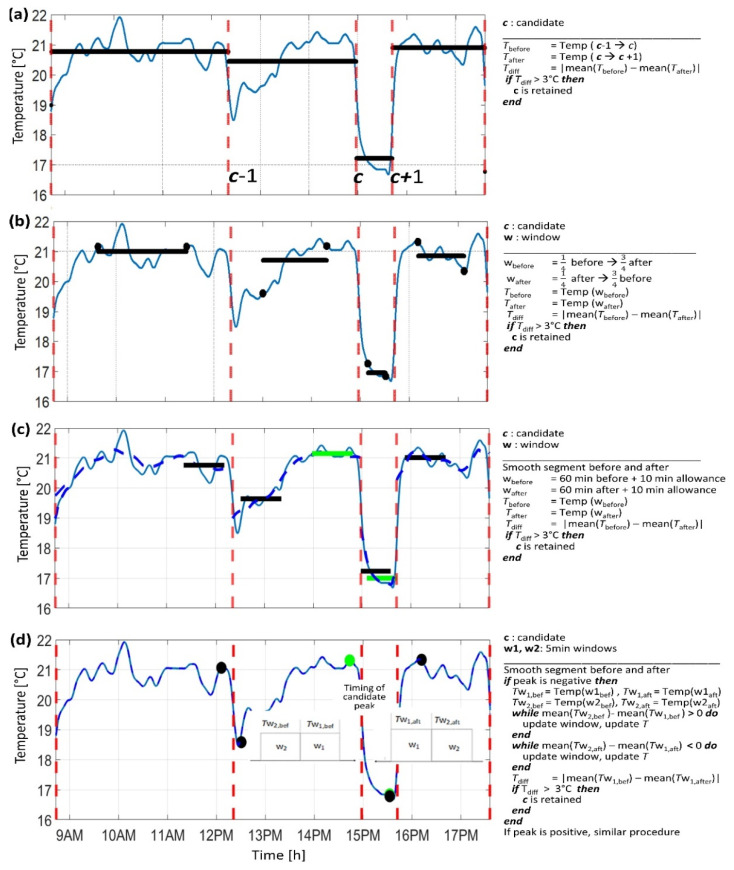
Analysis of the temperature signal around a candidate peak using four different approaches (**a**–**d**) for the selection of the signal segment to average the temperature level, estimate the level change, and skip or retain the candidate peak as an “event” associated with sensor removal/re-attachment. The dashed, red lines represent the candidates, and the horizontal lines and dots (black and green) represent the mean of the temperature over the windows adjacent to the candidate peak. The figure includes examples of the processed signal and the pseudocode corresponding to each method.

To analyze the surrounding of each candidate peak and classify/retain it as an “event” related to sensor removal/re-attachment, four options were tested. All options were based on a range centered on the evaluated candidate peak (*c*) that spanned from the previous (*c* − 1) to the following one (*c* + 1). The mean values of the signal over the window before (from *c*−1 to *c*) and after (from *c* to *c* + 1) were compared. Three of the options, illustrated in Figure 4a–c, were similar but varied in the length of the window used to compute the average temperature values as follows: (a) the entire signal segment between the evaluated, previous, and next candidate peak (Figure 4a); (b) the middle of the signal segment before and after the evaluated candidate peak (Figure 4b); (c) 60 min before and after the candidate peak, allowing 10 min to reach a steady state, according to the response time reported in the sensor datasheet (Figure 4c).

The fourth option (Figure 4d) was based on a different approach and used two bi-directional moving average windows (bi-moving windows) for estimating the trend of the temperature signal, similar to the approach proposed by Zhou et al. [3]. The average of the temperature signal was computed on the two windows simultaneously and then compared. The observed range was updated until a local maximum or a local minimum was reached. The procedure is exemplified in Figure 4d (see also pseudocode) by considering the timing of a negative candidate peak, potentially related to a sensor removal event. In the time interval before the peak timing, the bi-moving window was applied going back in time. As long as the mean value of temperature over window W1 was smaller than the mean over window W2 (i.e., the signal was decreasing towards the candidate), the windows were updated, meaning that W1 became W2 and W2 shifted backwards, then the averages were re-computed. Once the condition was no longer satisfied, a local maximum was reached, and it was taken as reference for the average temperature (mean(T_W1,bef_)). In the time interval after the peak timing, the same steps were applied but moving forward in time. Since the signal was decreasing away from the candidate, the mean over W2 was smaller than the mean over W1 until a local minimum was reached, and it was taken also as a reference for the average temperature (mean(T_W1,aft_)). For a positive candidate peak (potentially related to a reattachment event), the procedure was similar. If the difference, T_diff_ = |mean(T_W1,bef_) − mean(T_W1,aft_)|, was larger than 3 °C, the candidate was retained. The 3 °C threshold was chosen as optimal based on empirical observations of the data and a sensitivity analysis. Finally, the sign of the peak in the derivative signal was taken to classify the retained events as the start or the end of a non-wear period. The length of the window (W1, W2) was fixed at 5 min, which was considered an appropriate time interval to analyze the temperature without being influenced by small fluctuations. Note that the signal was further smoothed between candidates to remove small spurious fluctuation.

Note that after these steps, it was possible that a candidate was retained even though it was not related to an actual non-wear event. A subsequent “pruning process” was therefore applied in order to discard false positive detections. This procedure checked that every positive peak was followed by a negative one, and that the temperature remained low during non-wear. If this was not the case, the event was ignored.

#### 2.2.3. Combined Accelerometer and Temperature Based

Another possibility is to combine the output of the acceleration-based and temperature-based algorithms, as this approach can reduce the number of false negatives (i.e., periods that should be classified as wear are classified as non-wear). This was achieved by checking that both the acceleration and temperature conditions were satisfied at the same time, that is, the standard deviation of the acceleration vector magnitude (VM) and the temperature difference around the event were below their respective thresholds simultaneously.

All algorithms were implemented using MATLAB software (MATLAB2018b, MathWorks, Natick, MA, USA).

#### 2.2.4. Algorithm Validation Procedure

Data from five randomly selected participants were used to develop and optimize algorithm parameters, and data from the remaining 16 participants were used to estimate the algorithms’ performance. The ground truth was generated from the participant diaries where the times of sensor removal/re-attachment were carefully reported and used to create a MATLAB vector comparable to the output of algorithms. The ground truth contained labels for sensor “wear”, “non-wear”, and “worn in the pocket”.

The performances of the classifier were estimated in terms of sensitivity (SEN), specificity (SPE), negative predictive value (NPV), and accuracy (ACC). “Positive” events were related to wear detection and “negative” to non-wear, following the convention used by Zhou et al. [3]. Sensitivity and specificity indicated the proportion of actual wear and non-wear events (respectively) that were correctly identified by the algorithms. Negative predictive values expressed the proportion of non-wear data that the algorithm correctly identified as relevant [3]. Finally, accuracy evaluated the overall performance in classification.

These metrics were estimated for the data analysis results of each participant and reported as the mean (SD) for the group of 16 participants.

## 3. Results

### 3.1. Optimal Algorithm Parameters

**Figure 5:**
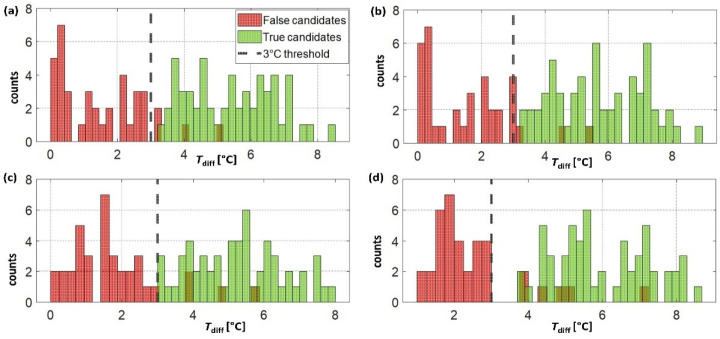
Distribution of the differences in temperature around the potential non-wear candidates for the four options of the event- and temperature-based algorithm. The red histograms are related to false and the green to true non-wear events. The dashed, vertical black line indicates 3 °C. The distributions were computed using the training data (five participants). In option 1 (**a**), option 2 (**b**), and option 3 (**c**), the candidates were discriminated based on the averages over the window spanning either the entire period between candidates, the period in the middle, or a 60 min interval, respectively, whereas in option 4 (**d**), averages were computed on a 5 min window around a local minimum/maximum.

Across the different algorithms, there were different hyperparameters to be estimated. For those inspired by state-of-the-art methods, the values were taken as proposed in current literature. For the new event- and temperature-based algorithms, the threshold used to discriminate true non-wear events was the most critical parameter to estimate. The value of 3 °C was chosen upon visual inspection after candidates were divided into true and false non-wear events. Indeed, this value was the most appropriate to discriminate candidates for all four approaches (see Figure 5).

### 3.2. Classification Performance

**Figure 6:**
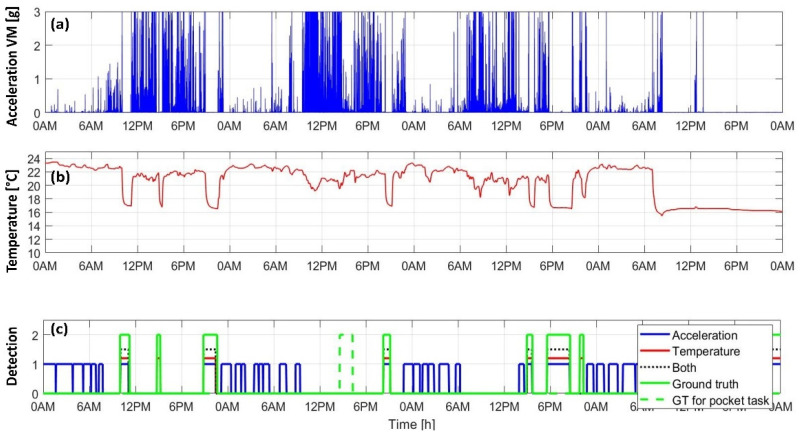
Illustrative example of processed sensor signals and the output of sensor non-wear detection algorithms: the filtered acceleration vector magnitude (**a**), the filtered and downsampled temperature signal (**b**), and the output of four different algorithms (based on accelerometer only, temperature only, or a combination of the two sensors) along with the ground truth extracted from participants’ diaries (**c**).

Figure 6 shows an illustrative example of the acceleration vector magnitude, VM, (Figure 6a), the smoothed temperature signal (Figure 6b), and the output of classification algorithms shown comparatively with the ground truth (Figure 6c). Periods of time where the sensor was carried near the body (i.e., in the pocket) are also highlighted. In this particular case, the event- and temperature-based algorithm with option 4 (Figure 4d) was used. Note that in this example, the second and last events were detected correctly by the temperature-based algorithm only.

**Figure 7:**
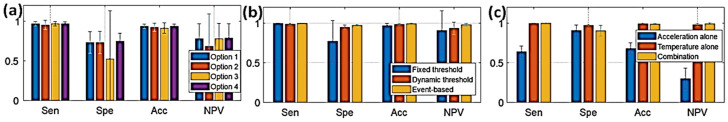
The mean and standard deviation of the sensitivity, specificity, accuracy, and NPV for the different algorithms. Panel (**a**) compares the performance of the four options of the event- and temperature-based algorithms; panel (**b**) compares the performance of window-based fixed and dynamic thresholds and event-based temperature algorithms; panel (**c**) compares the performance of algorithms based uniquely on the accelerometer, the temperature (event-based), or a combination of the two sensors.

Figure 7 shows comparatively the performances of all classification algorithms. As can be observed in Figure 7a, for the event- and temperature-based algorithms, option 4 resulted in slightly better performances, with 99%, 97%, 99%, and 98% for sensitivity, specificity, accuracy, and NPV, respectively. This algorithm also showed better performances when compared to state-of-the-art window- and temperature-based algorithms, both when using a fixed [3] and a dynamic [8] threshold as shown in Figure 7b. Similarly, it outperformed the algorithms using acceleration alone and the combination of acceleration and temperature (Figure 7c). The algorithm using the acceleration signal only showed the lowest performances (64%, 90%, 68%, and 30% for sensitivity, specificity, accuracy, and NPV, respectively), whereas the combination of the accelerometer and temperature signals showed a lower specificity (99%, 90%, 98%, and 98% for sensitivity, specificity, accuracy, and NPV, respectively) compared to temperature only. Furthermore, the proposed event- and temperature-based algorithm showed a lower standard deviation, suggesting good and consistent performances for all data sets (the 16 participants used for testing).

## 4. Discussion

The goal of the current study was to develop a robust algorithm that is able to distinguish between non-wearing and daily inactivity or (nighttime) sleep, as this is still an ongoing issue and can be problematic, especially when monitoring frail older adults or patients with severe conditions. The algorithm used a new approach based on specific events derived from the temperature signal, and a *relative* temperature threshold of 3 °C to discriminate true and false non-wear events. It was validated using real-world data and the performance was compared with state-of-the-art algorithms, showing high sensitivity and specificity.

Filtering and pre-processing: The performance of the event- and temperature-based algorithms depends on the peaks in the derivative of the temperature signal, with their amplitude being expected to increase with fast and important temperature changes, as, for example, when the device is removed and reattached. Due to the low signal-to-noise ratio and the slow response time of the temperature sensor, the amplitude of such peaks appeared very weak. Therefore, in order to enhance the relevant peaks, appropriate smoothing using the Savitzky-Golay filter and downsampling was necessary before applying the derivative operator.

Optimal parameter settings: The most critical parameters were those associated with the event- and temperature-based algorithms, especially the consideration of a minimum 3 °C difference in the temperature levels, which was necessary to classify the “event” as wear/non-wear (i.e., sensor attached/removed). The distribution of temperature differences before and after a peak in the derivative of the temperature signal indicated which value was more appropriate. In particular, the method using two bi-directional moving average windows (Figure 4d, option 4) showed a clearer distinction between false and actual non-wear (sensor removal) events (see Figure 5d). This suggests that this method is more suitable for estimating and discriminating between relevant temperature jumps in noisy fluctuations. Finally, the scanning windows estimated from the thermal response of the temperature sensor performed adequately. In particular, the 5 min window was able to accurately estimate the mean on a short window without being affected by noise (i.e., small variations).

Acceleration- vs. temperature-based methods: The common acceleration-based method appeared more prone to false positive errors compared to temperature-based algorithms. As can be observed in Figure 6, the acceleration-based algorithm frequently detected non-wear periods outside of the ranges covered by the ground truth vector. Moreover, those misclassified events often occurred during nighttime. The difficulty of finding an appropriate algorithm that can discriminate between sleep, sedentary behaviors, and non-wear periods has been highlighted in several studies [1,2,3,4,5,6,7,8,10]. The event- and temperature-based algorithms overcome this issue, as the classification depends on the proximity of the sensor to the body only. The temperature signal recorded during sleep and sedentary behaviors is characterized by higher values compared to non-wear, making the distinction easier and, thus, more reliable.

Regarding the temperature-based algorithms, the critical aspect affecting the performances was the exact detection of timing of the non-wear/wear onset and the delay associated with sensor response time. Due to the slow response time of the temperature sensor, the temperature derivative peaks appeared in the middle of a slope and not at the beginning nor end of the temperature decrease or increase. However, this accounts for small errors when classification is applied to long-term recorded sensor data. An additional critical aspect was the appropriate downsampling of the recorded temperature signal necessary to enhance the peaks in the derivative signal. For the Axivity device, the optimal downsampling (1/6) was selected based on the temperature sensor specifications (datasheet). Therefore, this might not be the optimal ratio for wearable devices including other types of temperature sensors. However, the algorithm could be easily adapted if sensor specifications are available.

When the output of acceleration-based and temperature-based algorithms were combined (“AND” operator), the performances were influenced by the inaccuracies of the acceleration-based algorithm. For an epoch to be classified as non-wear, it needed to be present in both cases. The useful aspect is that false positives that occur in one method or the other are ignored. The event-based temperature algorithm demonstrated encountering very few false positives and, in most of the cases, they were not corrected by the combination method. It would be interesting to investigate other aggregation (sensor fusion) methods in future studies.

As expected, the periods during which the sensor was worn in a different body location (e.g., in the pocket) were identified as “worn”, even by temperature-based algorithms due to the close vicinity to the body. As a general comment, most PA analysis algorithms consider that data are recorded at a specified body location; therefore, in such situations the pre-processing stage should include, in addition to non-wear, the detection of device location. Although interesting, the identification of how/where the device is worn during the monitoring period is a topic beyond the scope of the current study.

Progress beyond the state of the art: In Duncan et al.’s study [8], non-wearing detection was based on a dynamic threshold around 18 °C (*absolute temperature threshold*). As expected, when applied to the data set recorded in our study in a different geographic area (Norway in our study, New Zealand in [8]), the algorithm performance decreased considerably, especially in terms of specificity. The dynamic adjustment helped to shift the threshold to the middle of the temperature distribution (see Figure 2) and to distinguish better between low and high values. However, as the 18 °C starting point was not adapted to the current data, the new threshold was sometimes less optimal and captured some undesired fluctuations.

In more general terms, the use of a *relative temperature threshold* brings several potential advantages. The most important is that the algorithm may be more robust and more generalizable. An absolute threshold is specific to a particular region and the particular type of temperature sensor integrated into the monitoring device. For example, Zhou et al. [3] set a threshold at 26 °C using data recorded with a GENEActiv device, but this threshold was not appropriate for the Axivity device, as the absolute temperature range was lower. Both studies also pointed out that modifications were needed in order for the algorithms to work in different countries [3,8]. In contrast, the new method developed in the current study offers more flexibility and robustness, as it only depends on the relative temperature change. However, more extensive validation studies and data are necessary to confirm the generalizability of the results obtained in our study. Another interesting aspect is the increased specificity of our algorithm compared to the one used by Zhou et al. [3]. Indeed, the method described in [3] did not exceed ~65% specificity when the temperature algorithm was tested alone, compared to the 97% reached in the current study. This indicates that the event- and temperature-based algorithm with a relative threshold is more suited to identify non-wear transitions.

Future research perspectives: To ensure the quality of the validation data, the participants were healthy volunteers, having various daily activity patterns (active, sedentary), and were able to strictly follow the monitoring protocol (tasks execution, detailed diary). An extended validation of the algorithm(s) in other populations (e.g., frail older adults, chronic diseases/pain) might be interesting, provided that reliable ground truth data could be collected.

Clinical/epidemiological studies use various monitoring settings, for example, data recorded with various devices, on different body segments (lower back, chest, wrist, thigh, and ankle) and fixation modalities (e.g., on skin, belt, watch). Therefore, an important perspective of the current study is to evaluate the performances of temperature-based non-wear detection algorithms on data recorded in such alternative settings.

Finally, the algorithms described use an approach based on signal processing and data-driven decision rules. Alternative methods, using features extracted from the temperature/acceleration signals and machine learning-based classifiers [10] could be investigated as well in future studies, if larger amounts of data (with the ground truth) are available for development of the classification models.

## 5. Conclusions

Several new and state-of-the-art algorithms for detection of non-wearing of wearable devices were implemented and evaluated. Data recorded with the temperature sensor integrated into the monitoring device (Axivity), combined with appropriate signal processing methods, allowed for the most accurate identification of the periods where the device was removed. The classical approach based on the acceleration signal showed very low performances for data collected in our study, especially in terms of sensitivity, accuracy, and NPV (64%, 68%, and 30%, respectively), whereas the event- and temperature-based algorithm showed a significant improvement (99%, 99%, and 98%, respectively). These results open up new perspectives for accurate/unbiased assessment of long-term PA, given that the trend of wearable technologies is to integrate additional sensor modalities in addition to the inertial sensors (accelerometers, gyroscopes).

## Data Availability

The data presented in this study are available upon request from the corresponding author.
